# A comprehensive analysis of the association of psychological profiling and sagittal balance parameters with neck pain in a non-deformity cervical spondylosis population: A prospective two-centers cross-sectional study

**DOI:** 10.1007/s10143-026-04527-z

**Published:** 2026-10-19

**Authors:** Marco Battistelli, Sofia Vannuccini, Massimo Benenati, Mario Muselli, Nicola Montano, Massimiliano Visocchi, Gabriele Capo, Carlo Brembilla, Maurizio Fornari, Simona Gaudino, Francesco Doglietto, Filippo Maria Polli, Daniela Pia Rosaria Chieffo

**Affiliations:** 1 Medicine and Surgery, Catholic University of the Sacred Heart, Rome, Italy; 2 Neurosurgery, Fondazione Policlinico Universitario Agostino Gemelli IRCCS, Largo A. Gemelli, 8, Rome, 00168 Italy; 3 Clinical Psychology Unit, Fondazione Policlinico Universitario Agostino Gemelli IRCCS, Rome, Italy; 4 Dipartimento Di Diagnostica Per Immagini, Fondazione Policlinico Universitario A. Gemelli IRCCS, Radioterapia, Rome, Oncologia Ed Ematologia Italy; 5 Department of Life, Health and Environmental Sciences, University of L’Aquila, L’Aquila, Italy; 6 Department of Neurosurgery, IRCCS Humanitas Research Hospital, Milan, 20089 Italy

**Keywords:** Cross-sectional study, Neck pain, Cervical sagittal balance, Psychological profiling factors, Cervical spondylosis, Symptom Checklist-27 (SCL-27)

## Abstract

Purpose to comprehensively identify determining factors of perceived neck pain in a non-deformity population, integrating psychological and sagittal balance parameters.

Methods a cross-sectional study of prospectively collected data was conducted on patients admitted for cervical spondylosis at two tertiary university hospitals between April 2024 and August 2025. Eligible cases were retrieved from the PANDA Registry. Records were excluded if standing X-ray was unavailable and/or not-evaluable (e.g. shoulder prosthesis); or if the admission pathology involved the cranio-cervical junction, tumors, deformities, spinal cord disease, or infections. Preoperative data included demographic, clinical (VAS neck), psychological (SCL-27, I-TIPI, CD-RISC10), and sagittal balance variables (McGregor slope, C1–C2 lordosis, C2 slope, C2–C7 lordosis, cSVA, T1 slope, cervical mismatch). Correction for multiple comparisons was applied within pre-specified hypothesis domains.

Results one-hundred-thirty records were included. Intra-rater reliability for radiographic parameters was excellent (> 0.9). After categorization of patients by neck pain severity, pairwise analysis showed that only C1–C2 lordosis remained significantly higher in patients exhibiting severe neck pain (VAS < 7 vs. VAS ≥ 7). When stratified for moderate-to-severe neck pain (VAS ≥ 4), several SCL-27 scales, namely dysthymic, vegetative, agoraphobic, sociophobic, and GSI scores, and C1C2 lordosis showed significant differences. Correlation analysis using VAS neck as the dependent variable demonstrated a significant positive correlation with SCL-27 dysthymic and vegetative scores. C1–C2 lordosis showed a significant inverse correlation with neck pain. In univariate analysis, only SCL-27 dysthymic, vegetative, and GSI scores emerged as significant univariate associations of VAS neck. After correction for multiple comparisons, the SCL-27 vegetative, dysthymic and GSI scores retained significance, whereas C1–C2 lordosis did not.

Conclusion in a non-deformity population, sagittal balance parameters alone do not adequately explain patient-perceived neck pain. These findings support the preoperative use of psychological assessments, especially the SCL-27, as a useful tool in preoperative screening and individualised counselling.

**Supplementary Information:**

The online version contains supplementary material available at https://doi.org/10.1007/s10143-026-04527-z.

## Introduction

The pathophysiology of neck pain has been mostly investigated focusing on degenerative spondylotic changes of the intervertebral discs and/or facet joints and their consequences over cervical mechanics. The ISSG 2015 classification system highlighted three key parameters of cervical alignment: the magnitude of cervical lordosis (CL) in relation to the T1 slope (T1S), the cervical sagittal vertical axis (cSVA), and the ability to maintain horizontal gaze, described by the chin–brow vertical angle [1]. Although primarily developed for cervical deformity, this classification system redirected research towards mechanical explanations of neck pain. Consequently, several sagittal balance parameters—CL, cSVA, T1S, and the cervical mismatch (T1S–CL)—have been associated with neck pain and related disability [2, 3]. However, other authors have reported conflicting results. Fujiwara et al. did not demonstrate an association between neck pain and preoperative or postoperative radiographic cervical alignment [4]. These discrepancies may partly reflect an incomplete understanding of cervical biomechanics, including the role of cervical curvature in relation to the cervicothoracic “base” and the compensatory mechanisms involving cSVA, as well as heterogeneity in the studied populations regarding the presence of deformity [5–7].

Uncertainty in understanding neck pain is partly due to the still limited number of studies that have systematically investigated the role of psychological factors in its genesis and maintenance [8–11]. The definition proposed by the International Association for the Study of Pain emphasizes that pain is a complex experience, influenced not only by nociceptive processes but also by emotional, cognitive, and contextual factors, which substantially contribute to its perception and chronicity [12]. From this perspective, neck pain cannot be interpreted exclusively as an expression of structural alterations, but as the result of a dynamic interaction between biological and psychological dimensions.

Numerous findings from the literature on musculoskeletal and spinal pain indicate that psychological distress plays a central role in modulating pain intensity and perceived disability. In particular, the presence of depressive symptoms, emotional distress, and alterations in affective balance has been associated with increased pain perception, poorer overall functioning, and less favourable clinical outcomes, regardless of the severity of documented structural alterations [13]. This evidence suggests that psychological factors may act as amplifiers of the pain experience, contributing to its persistence over time. Psychological therapies are among the recommended treatments for neck pain [14, 15]. Adaptation to stress and resilience are linked to less pain-related disability, and a better response to conservative treatments, while poor coping skills and higher psychological vulnerability are associated with more severe pain and greater functional impairment [16]. Recent research on chronic low back pain has shown a relationship between pain intensity and both psychological and sagittal factors [17, 18]. Although this evidence comes from studies on the lumbar spine, it supports the hypothesis that the interaction between biomechanical and psychological factors may influence pain perception in the cervical spine.

In consideration of these gaps in the literature, we investigated the origin of neck pain in a non-deformity cohort of patients with cervical spondylosis. We performed a comprehensive assessment of sagittal balance parameters and psychological factors. Our aims were to determine: (1) the relationship between reported neck pain, sagittal parameters, and psychological factors; (2) how these factors differ between neck pain severity groups; and (3) predictors of neck pain in the overall cohort and in subgroups stratified by pain severity.

## Materials and methods

**Fig. 1:**
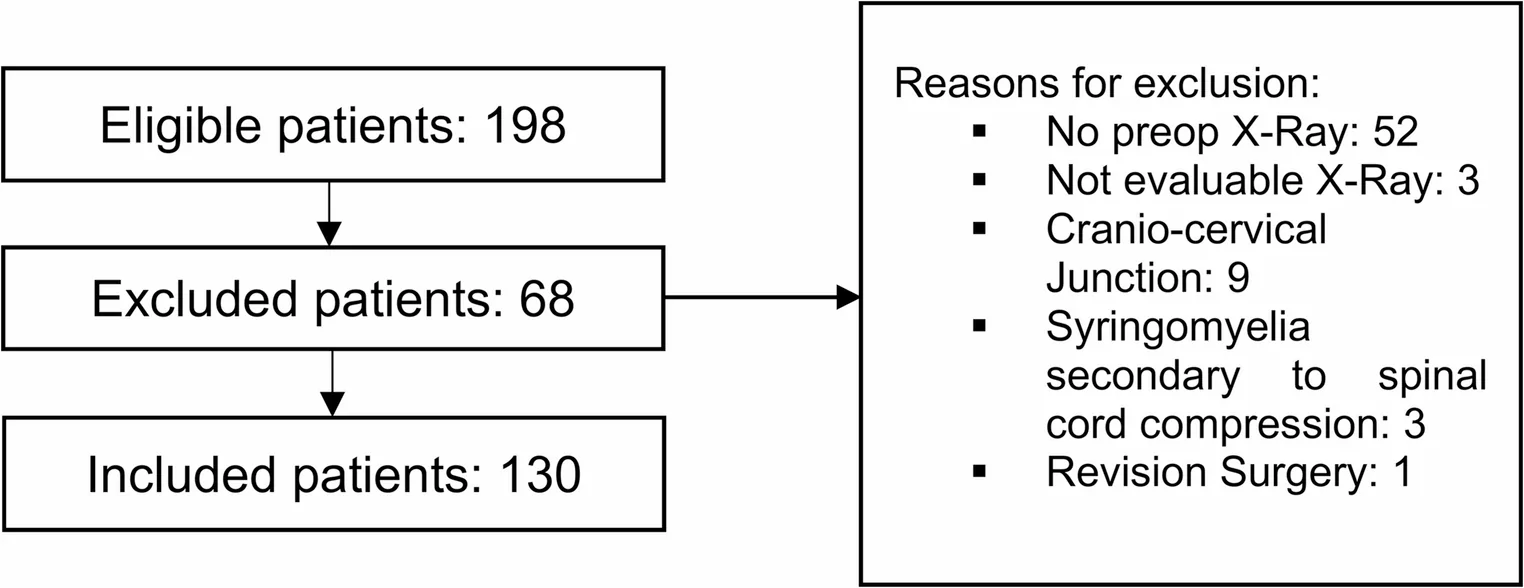
Records’ selection flow-chart

A cross-sectional analysis of prospectively registered data was conducted on medical records of patients admitted for cervical spondylosis at two tertiary university hospitals between April 2024 and August 2025. Exclusion criteria were absence of a preoperative standing X-ray and/or non-assessable X-ray (e.g. shoulder prosthesis); pathology involving the cranio-cervical junction; admission for tumors, deformities, intrinsic spinal cord pathology (intramedullary lesions and secondary syringomyelia), and/or infections; revision surgery. Patients affected by degenerative cervical myelopathy were included, whereas those affected by a diagnosed psychiatric disorder were excluded. The inclusion flow-chart is shown in Fig. 1. Patients’ records identification and data extraction were performed using the prospective Progressive Advancement in Neurosurgery: Data and Analysis (PANDA) Registry, which was approved by the local IRB (Comitato Etico Lazio (CET) 3, ID: 7202) and registered on clinicaltrials.gov (NCT06835634, date of registration 15/01/2025). Every patient provided an informed consent to data collection and publication. This study is reported according to the STROBE guidelines and in accordance with the ethical principles of the Declaration of Helsinki.

### Outcomes and collected parameters

The primary outcome of this study was a comprehensive assessment of the determinants of perceived neck pain, focusing on previously described sagittal balance parameters and a set of psychological measures defined during a multidisciplinary meeting with the Clinical Psychology Department of our hospital. Neck pain intensity (VAS Neck) was obtained from the item “How severe was your neck pain in the last week?” of the COMI Neck questionnaire. Collected variables included demographic factors (age, BMI, smoking status, Charlson Comorbidity Index, and Frailty Index), VAS Neck, psychological scales (Symptom Checklist-27 [SCL-27], Italian Ten-Item Personality Inventory [I-TIPI], Connor–Davidson Resilience Scale – 10 items [CD-RISC10]) and sagittal balance parameters from standing X-rays (C1–C2 lordosis [UCL], C2–C7 lordosis [CL], T1 slope [T1S], cervical mismatch [T1S–CL], cervical SVA [cSVA], McGregor slope [MGS], C2 slope [C2S]). By convention, lordotic angles were assigned negative values, while kyphotic angles were assigned positive values. Sagittal balance parameters were measured independently by a spine fellow (M.B.) and a senior spine-specialized radiologist (M.B.), who were blinded to patients’ demographic, clinical and psychological data.

### Psychological scales description

To assess how emotional distress, resilience, and personality traits contribute to neck pain perception, three validated psychometric instruments were administered.

Psychological symptomatology was evaluated using the Symptom Checklist-27 (SCL-27), a multidimensional tool specifically developed and validated for use with chronic pain populations, including those with spinal conditions. Its structure allows efficient identification of depressive, dysthymic, vegetative, agoraphobic, and social anxiety symptoms, in addition to mistrust-related features [16]. The instrument’s sensitivity to both emotional and somatic distress is particularly pertinent for patients with cervical degenerative pathology, whose symptom experiences often reflect intertwined physical and psychological processes.

Resilience was assessed using the Connor–Davidson Resilience Scale (CD-RISC-10). This short form has shown robust psychometric properties and cross-cultural applicability [19, 20]. The instrument is self-administered, and respondents indicate their agreement with each item on a 5-point Likert scale ranging from 0 (“not true at all”) to 4 (“true nearly all the time”), producing total scores ranging from 0 to 40, where higher scores indicate greater resilience.

Personality traits were measured using the Italian Ten-Item Personality Inventory (I-TIPI), which assesses the Big Five domains in a brief, clinically practical format [21, 22]. Each of the 10 items consists of two opposing descriptors. At the beginning of the tool, there is the phrase “I see myself as.” to respond to the items based on this statement. The respondents rate their agreement on a 7-point Likert scale from 1 (“strongly disagree”) to 7 (“strongly agree”). The Italian version (I-TIPI) was adapted and validated by Chiorri et al., maintaining the essential psychometric properties for a rapid and reliable assessment of personality [21]. Although its brevity entails reduced granularity compared to longer personality measures, the I-TIPI reliably captures emotional stability, extraversion, agreeableness, conscientiousness, and openness.

### Statistical analysis

The statistical analysis for this study used several complementary methods to evaluate the relationships between neck pain, psychological factors, and radiographic parameters. First, a descriptive analysis was performed, with continuous variables summarized as mean ± standard deviation and categorical variables as frequencies and percentages. Inter-rater reliability for radiographic measurements was assessed using concordance coefficients. The mean values were used for the subsequent analysis.

In line with the primary aims of the study, (1) to examine the relationship between reported neck pain, sagittal parameters, and psychological factors, Spearman’s rank correlation was used to calculate the Rho coefficient and its statistical significance. To address (2) how sagittal and psychological parameters differ between neck pain intensity groups, the sample was stratified into categories based on VAS scores to identify differences in psychological status and/or sagittal balance across pain severity levels. The Mann–Whitney U test was applied for pairwise comparisons between two groups (VAS Neck ≤ 6 vs. VAS Neck ≥ 7; and VAS Neck ≤ 3 vs. VAS Neck ≥ 4). Finally, univariate regression analyses were performed for the total cohort to quantify the effect of individual psychological and radiographic parameters.

The Benjamini–Hochberg procedure was applied to control the false discovery rate at 0.05, and adjusted p-values (q-values) are reported alongside nominal p-values [23]; Bonferroni-adjusted p-values are additionally reported as a more conservative sensitivity check. Correction was applied separately to each analytical family (correlation analysis, each pairwise subgroup comparison, and univariate regression).

The self-administered psychological questionnaires were returned incomplete by a proportion of patients (Table 1). Missing data were not imputed in the primary analysis, which was performed on complete cases. Because questionnaire incompleteness was found to be associated with neck pain intensity, a sensitivity analysis based on multivariate imputation by chained equations was performed. Twenty imputed datasets were generated using all psychological, radiographic and demographic variables together with the outcome as predictors, with imputed values constrained within the admissible range of each instrument; the Global Severity Index was derived passively from the imputed subscale scores. VAS Neck was never imputed. Estimates were pooled according to Rubin’s rules, with correlation coefficients pooled on the Fisher z scale, and are reported in Supplementary Table S1.

**Table 1:** Completeness of self-administered psychological questionnaires according to neck pain severity

Neck pain VAS	*n*	SCL-27 complete, *n* (%)	I-TIPI complete, *n* (%)	CD-RISC-10 complete, *n* (%)
VAS Neck ≤ 3	43	42 (97.6)	42 (97.6)	41 (95.3)
VAS Neck 4–6	36	31 (86.1)	31 (86.1)	31 (86.1)
VAS Neck ≥ 7	51	38 (74.5)	43 (84.3)	45 (88.2)
Overall	130	111 (85.4)	116 (89.2)	117 (90.0)

The proportion of patients with an incomplete SCL-27 increased monotonically with neck pain severity (Cochran-Armitage test for trend, z = 3,17, *p* = 0.0015). Patients with an incomplete SCL-27 reported significantly higher neck pain than those with complete data (6.9 ± 2.3 vs. 4.7 ± 3.1; Mann-Whitney U test, *p* = 0.006). No radiographic parameter, nor age, body mass index or Charlson Comorbidity Index, differed between patients with complete and incomplete questionnaires (all *p* > 0.2). *SCL-27* Symptom checklist-27, *I-TIPI* Ten-item personality inventory, *CD-RISC10* Connor-davidson resilience scale − 10 items, *VAS* Visual analogue scale

## Results

Of the 198 potentially eligible records, 68 were excluded after application of exclusion criteria. Eventually, 130 records were included for analysis (Fig. 1). The included population presented a mean age of 58.8 ± 12.2, a mean age-adjusted Charlson Comorbidity Index of 3.1 ± 2.2 and a mean VAS neck of 5.0 ± 3.1. Of note, radiographic parameters showed a population without significant alterations in the sagittal parameters, with a mean cSVA of 27.9 ± 13.6 mm and a mean cervical mismatch of 19.1 ± 10.6°. The psychological assessment provides an overview of psychological symptoms, personality traits, and resilience in patients with neck pain. SCL-27 scores showed mean values of 0.5 ± 0.4 for depressive symptoms, 0.9 ± 0.8 for dysthymic symptoms, 0.5 ± 0.6 for vegetative symptoms, 0.3 ± 0.4 for agoraphobic symptoms, 0.3 ± 0.5 for sociophobic symptoms, and 0.5 ± 0.6 for mistrust. The mean Global Severity Index (GSI) was 0.5 ± 0.4, indicating overall low-to-moderate levels of psychological symptomatology with relatively homogeneous distributions across symptom domains. Personality trait scores were 4.2 ± 1.4 for Extraversion, 5.3 ± 1.1 for Agreeableness, 5.5 ± 1.2 for Conscientiousness, 4.1 ± 1.2 for Openness to Experience, and 4.6 ± 1.3 for Emotional Stability (Table 2). Compared with normative data, agreeableness and conscientiousness appeared slightly higher, while extraversion, openness, and emotional stability fell within expected reference ranges. Overall, the personality profile of the cohort did not show clinically relevant deviations from population norms.

**Table 2:** Descriptive analysis

Age	58.8 ± 12.2
BMI (Kg/M^2^)	26.1 ± 4.3
Smoking Habit	
No	83 (63.8%)
Yes	27 (20.8%)
Ex-Smoker	20 (15.4%)
Charlson Comorbidity Index	
** • **Hypertension	
No	93 (71.5%)
Yes	37 (28.5%)
• Previous Myocardial Infarction	
No	121 (93.1%)
Yes	9 (6.9%)
• Heart Failure	
No	126 (96.9%)
Yes	4 (3.1%)
• Cerebrovascular Disease	
No	127 (97.7%)
Yes	1 (0.8%)
Yes, Previous	2 (1.5%)
• Peripheral Vascular Disease	
No	126 (96.9%)
Yes	4 (3.1%)
• Chronic Lung Disease	
No	126 (96.9%)
Yes	4 (3.1%)
• Gastric Or Duodenal Ulcer	
No	125 (96.2%)
Yes	1 (0.8%)
Yes, Previous	4 (3.1%)
• Mild Liver Disease	
No	126 (96.9%)
Yes	3 (2.3%)
Yes, Previous	1 (0.8%)
• Severe Liver Disease	
No	129 (99.2%)
Yes	1 (0.8%)
• Renal Disease	
No	123 (94.6%)
Yes	4 (3.1%)
Yes, Previous	3 (2.3%)
• Rheumatic Disease	
No	127 (97.7%)
Yes	3 (2.3%)
• Dementia	
No	129 (99.2%)
Yes	1 (0.8%)
• Lymphoma	
No	127 (97.7%)
Yes	1 (0.8%)
Yes, Previous	2 (1.5%)
• Leukemia	
No	130 (100.0%)
• Any Malignancy	
No	123 (94.6%)
Yes	1 (0.8%)
Yes, Previous	6 (4.6%)
• Metastatic Solid Tumor	
No	128 (98.5%)
Yes, Previous	2 (1.5%)
• Acquired Immunodeficiency Syndrome	
No	128 (98.5%)
Yes	1 (0.8%)
Yes, Previous	1 (0.8%)
• Diabetes	
No	117 (90.0%)
Yes	13 (10.0%)
• Hemiplegia	
No	130 (100.0%)
• Other Pathologies	
No	107 (82.3%)
Yes	17 (13.1%)
Yes, Previous	6 (4.6%)
• Age-Adjusted CCI	3.1 ± 2.2
Frailty Index	0.5 ± 0.7
VAS Neck	5.0 ± 3.1
SCL-27	
• Depressive Score	0.5 ± 0.4
• Dysthymic Score	0.9 ± 0.8
• Vegetative Score	0.5 ± 0.6
• Agoraphobic Score	0.3 ± 0.4
• Sociophobic Score	0.3 ± 0.5
• Mistrust Score	0.5 ± 0.6
• GSI	0.5 ± 0.4
I-TIPI	
• Extraversion	4.2 ± 1.4
• Agreeableness	5.3 ± 1.1
• Conscientiousness	5.5 ± 1.2
• Openness to Experience	4.1 ± 1.2
• Emotional Stability	4.6 ± 1.3
CD-RISC-10	
• Connor-Davidson Scale	25.5 ± 6.2
Sagittal Balance Parameters	
• C1C2 lordosis (°)	-30.6 ± 8.1
• C2C7 lordosis (°)	-8.4 ± 12.6
• T1S (°)	27.5 ± 8.7
• T1-CL (°)	19.1 ± 10.6
• cSVA (mm)	27.9 ± 13.6
• MGS (°)	-1.4 ± 7.5
• C2S (°)	17.8 ± 9.2

*BMI* Body mass index, *CCI* Charlson comorbidity index, *VAS* Visual analogue scale, *SCL-27* Symptom checklist-27, *I-TIPI* Ten-item personality inventory, *CD-RISC10* Connor-davidson resilience scale − 10 items, *T1S (°)* T1 slope, *cSVA* Cervical sagittal vertical axis,* C2S (°)* C2 slope, *MGS (°)* McGregor slope

The mean CD-RISC-10 score in the study population was 25.5 ± 6.2, which is lower than values typically reported in normative samples of the general population, usually ranging between approximately 29 and 33 points. Compared with these reference values, the present cohort showed reduced resilience levels, while remaining within the overall variability observed in community-based samples (Table 1). Inter-rater agreement showed excellent reliability of all sagittal balance measurements (Concordance Coefficient > 0.9) (Table 3).

**Table 3:** Inter-rater agreement between measurements of sagittal balance parameters

C1C2 lordosis (°)	Concordance Coefficient	0.948
	p-value	< 0.001
C2C7 lordosis (°)	Concordance Coefficient	0.953
	p-value	< 0.001
T1S (°)	Concordance Coefficient	0.93
	p-value	< 0.001
T1-CL (°)	Concordance Coefficient	0.948
	p-value	< 0.001
cSVA (mm)	Concordance Coefficient	0.981
	p-value	< 0.001
MGS (°)	Concordance Coefficient	0.931
	p-value	< 0.001
C2S (°)	Concordance Coefficient	0.919
	p-value	< 0.001

T1 Slope, *cSVA* Cervical sagittal vertical axis, *C2S (°)* C2 slope, *MGS (°)* McGregor slope

### Correlation between reported neck pain, sagittal parameters, and psychological factors

Correlation analysis documented a significant correlation between perceived neck pain and several psychological parameters. Namely, dysthymic (*R* = 0.239; p-value = 0.015) and vegetative (*R* = 0.344; p-value < 0.001) subscores of SCL-27 exhibited a significant correlation. Also, GSI exhibited a tendency towards significance (*R* = 0.198; p-value = 0.053). Of note, none of the resilience and personality items showed correlation with perceived neck pain. Among radiographic parameters, C1C2 lordosis exhibited a significant negative correlation with perceived neck pain (*R* = -0.244; p-value = 0.01) (Table 4).

**Table 4:** Correlation between VAS Neck and psychological parameters, and radiographic parameters

Dependent variable: VAS neck		
SCL-27		
Depressive Score	Rho di Spearman	0.054
	p-value	0.588
Dysthymic Score	Rho di Spearman	0.239
	p-value	**0.015**
Vegetative Score	Rho di Spearman	0.344
	p-value	**< 0.001**
Agoraphobic Score	Rho di Spearman	0.155
	p-value	0.120
Sociophobic Score	Rho di Spearman	0.079
	p-value	0.437
Mistrust Score	Rho di Spearman	-0.056
	p-value	0.578
GSI	Rho di Spearman	0.198
	p-value	0.053
I-TIPI		
Extraversion	Rho di Spearman	0.014
	p-value	0.889
Agreeableness	Rho di Spearman	-0.120
	p-value	0.223
Conscientiousness	Rho di Spearman	-0.017
	p-value	0.865
Openness to Experience	Rho di Spearman	0.048
	p-value	0.632
Emotional Stability	Rho di Spearman	-0.017
	p-value	0.867
CD-RISC-10		
Connor-Davidson Scale	Rho di Spearman	-0.076
	p-value	0.452
Sagittal Balance Parameters		
C1C2 lordosis (°)	Rho di Spearman	-0.244
	p-value	**0.010**
C2C7 lordosis (°)	Rho di Spearman	0.055
	p-value	0.563
T1S (°)	Rho di Spearman	0.006
	p-value	0.948
T1-CL (°)	Rho di Spearman	0.151
	p-value	0.109
cSVA (mm)	Rho di Spearman	0.076
	p-value	0.424
MGS (°)	Rho di Spearman	-0.114
	p-value	0.236
C2S (°)	Rho di Spearman	0.136
	p-value	0.151

*SCL-27* Symptom checklist-27, *I-TIPI* Ten-item personality inventory, *CD-RISC10* Connor-davidson resilience scale − 10 items, *T1S (°)* T1 slope, cSVA Cervical Sagittal vertical axis *C2S (°)* C2 slope; *MGS (°)* McGregor slope. Bold font indicates statistical significance

### Differences in sagittal alignment and psychological factors in perceived neck pain severity groups

When comparing patients reporting mild versus moderate-to-severe neck pain, most SCL-27 subscales differed significantly between groups. In particular, dysthymic (0.7 ± 0.6 vs. 1.1 ± 0.8; *p* = 0.006), vegetative (0.2 ± 0.2 vs. 0.7 ± 0.7; *p* < 0.001), agoraphobic (0.2 ± 0.3 vs. 0.4 ± 0.4; *p* = 0.029), sociophobic (0.2 ± 0.4 vs. 0.4 ± 0.6; *p* = 0.043), and GSI (0.3 ± 0.3 vs. 0.6 ± 0.4; *p* = 0.008) subscale scores were significantly higher in the VAS ≥ 4 group. Among sagittal parameters, C1–C2 angle was more lordotic in the moderate-to-severe neck pain group (-28.3 ± 6.7 vs. -30.9 ± 8.8; *p* = 0.022). Conversely, in a pairwise analysis stratified for severe neck pain (VAS < 7 vs. VAS ≥ 7), only C1–C2 lordosis remained significantly more pronounced in the group with more severe neck pain (− 29.1 ± 7.8 vs. −31.6 ± 8.6; *p* = 0.024) (Table 5).

**Table 5:** Pairwise comparisons of light versus moderate-to-severe perceived neck pain

	VAS neck ≤ 3	VAS neck ≥ 4	*p*-value	VAS neck ≤ 6	VAS neck ≥ 7	*p*-value
SCL-27						
Depressive Score	0.4 ± 0.4	0.5 ± 0.5	0.324	0.5 ± 0.4	0.5 ± 0.4	0.832
Dysthymic Score	0.7 ± 0.6	1.1 ± 0.8	**0.006**	0.9 ± 0.8	1.0 ± 0.7	0.318
Vegetative Score	0.2 ± 0.2	0.7 ± 0.7	**< 0.001**	0.4 ± 0.5	0.6 ± 0.7	0.106
Agoraphobic Score	0.2 ± 0.3	0.4 ± 0.4	**0.029**	0.3 ± 0.4	0.3 ± 0.4	0.754
Sociophobic Score	0.2 ± 0.4	0.4 ± 0.6	**0.043**	0.3 ± 0.5	0.3 ± 0.5	0.752
Mistrust Score	0.4 ± 0.5	0.5 ± 0.6	0.745	0.6 ± 0.6	0.4 ± 0.5	0.063
GSI	0.3 ± 0.3	0.6 ± 0.4	**0.008**	0.5 ± 0.4	0.5 ± 0.4	0.988
I-TIPI						
Extraversion	4.4 ± 1.5	4.2 ± 1.3	0.383	4.1 ± 1.5	4.4 ± 1.3	0.461
Agreeableness	5.4 ± 1.1	5.3 ± 1.1	0.473	5.5 ± 1.1	5.1 ± 1.2	0.173
Conscientiousness	5.4 ± 1.2	5.5 ± 1.2	0.845	5.5 ± 1.2	5.4 ± 1.2	0.940
Openness to Experience	4.2 ± 1.0	4.1 ± 1.3	0.897	4.1 ± 1.2	4.1 ± 1.2	0.901
Emotional Stability	4.9 ± 1.3	4.4 ± 1.4	0.150	4.5 ± 1.4	4.8 ± 1.3	0.445
CD-RISC-10						
Connor-Davidson Scale	26.8 ± 4.5	24.8 ± 6.8	0.116	25.6 ± 5.9	25.3 ± 6.7	0.675
Sagittal Balance						
C1C2 lordosis (°)	-28.3 ± 6.7	-30.9 ± 8.8	**0.022**	-29.1 ± 7.8	-31.6 ± 8.6	**0.024**
C2C7 lordosis (°)	-6.7 ± 12.4	-8.3 ± 12.7	0.675	-8.7 ± 12.2	-6.2 ± 13.1	0.281
T1S (°)	26.1 ± 9.9	27.5 ± 7.7	0.461	27.6 ± 8.8	26.3 ± 7.9	0.313
T1-CL (°)	19.3 ± 12.6	19.4 ± 9.7	0.367	18.9 ± 11.0	20.2 ± 10.3	0.247
cSVA (mm)	28.1 ± 15.1	27.4 ± 13.3	0.830	26.9 ± 14.0	28.7 ± 13.7	0.554
MGS (°)	0.0 ± 6.5	-1.7 ± 7.9	0.101	-0.8 ± 7.5	-1.7 ± 7.5	0.212
C2S (°)	17.7 ± 9.3	18.2 ± 9.2	0.707	17.3 ± 8.9	19.2 ± 9.6	0.280

*SCL-27* Symptom checklist-27, *I-TIPI* Ten-item personality inventory, *CD-RISC10*: Connor-davidson resilience scale − 10 items, *T1S (°)* T1 slope; *cSVA* Cervical sagittal vertical axis, *C2S (°)* C2 Slope, *MGS (°)* McGregor slope. Bold font indicates statistical significance

### Univariate associations of neck pain

When analysing univariate associations of self-reported neck pain in the overall study population, only scores from the SCL-27 emerged as statistically significant. Specifically, dysthymic (ß-coeff.= 0.99, 95% CI 0.19–1.79; *p* = 0.016), vegetative (ß-coeff.= 1.81, 95% CI 0.83–2.79; *p* < 0.001), and GSI (ß-coeff.= 1.81, 95% CI 0.25–3.37; *p* = 0.024) scores were associated with neck pain severity. Notably, C1–C2 lordosis no longer showed a statistically significant association in this analysis (*p* = 0.094) (Table 6).

**Table 6:** Univariate regression with VAS Neck as dependent variable and psychological parameters, and radiographic parameters as independent variables

	ß-coefficient	95% Confidence Interval		*p*-value
SCL-27				
Depressive Score	0.25	-1.16	1.66	0.729
Dysthymic Score	0.99	0.19	1.79	**0.016**
Vegetative Score	1.81	0.83	2.79	**< 0.001**
Agoraphobic Score	1.22	-0.31	2.75	0.117
Sociophobic Score	0.52	-0.71	1.76	0.402
Mistrust Score	-0.03	-1.13	1.07	0.954
GSI	1.81	0.25	3.37	**0.024**
I-TIPI				
Extraversion	0.03	-0.42	0.48	0.895
Agreeableness	-0.31	-0.87	0.25	0.269
Conscientiousness	-0.05	-0.57	0.47	0.858
Openness to Experience	0.05	-0.49	0.59	0.859
Emotional Stability	-0.12	-0.59	0.34	0.608
CD-RISC-10				
Connor-Davidson Scale	-0.04	-0.15	0.06	0.393
Sagittal Balance Parameters				
C1C2 lordosis (°)	-0.06	-0.13	0.01	0.094
C2C7 lordosis (°)	0	-0.04	0.05	0.918
T1S (°)	0.01	-0.05	0.08	0.677
T1-CL (°)	0.02	-0.04	0.07	0.575
cSVA (mm)	0.01	-0.03	0.06	0.530
MGS (°)	-0.03	-0.11	0.05	0.457
C2S (°)	0.04	-0.03	0.1	0.255

*SCL-27* Symptom checklist-27,* I-TIPI* Ten-item personality inventory, *CD-RISC10* Connor-davidson resilience scale − 10 items, *T1S (°)* T1 slope, *cSVA* Cervical sagittal vertical axis, *C2S (°)* C2 slope, *MGS (°)* McGregor slope. Bold font indicates statistical significance

### Sensitivity analysis

After Benjamini–Hochberg correction within hypothesis domains, the SCL-27 vegetative subscale remained significant in all analyses (correlation q = 0.006; VAS ≤ 3 vs. ≥ 4 comparison q = 0.006; univariate regression q = 0.006). In the comparison between mild and moderate-to-severe neck pain, the dysthymic subscale (q = 0.035) and the GSI (q = 0.035) also remained significant, whereas the agoraphobic (q = 0.094) and sociophobic (q = 0.112) subscales did not. Among radiographic parameters, C1–C2 lordosis did not retain significance after correction in any analysis (correlation q = 0.070; VAS ≤ 3 vs. ≥ 4 q = 0.154; VAS ≤ 6 vs. ≥ 7 q = 0.168; univariate regression q = 0.658). Under the Bonferroni correction, only the vegetative subscale remained significant. Adjusted p-values for all nominally significant results are reported in Table 7.

**Table 7:** Correction for multiple comparisons: nominal and adjusted p-values for all results reaching nominal statistical significance

Variable	Hypothesis domain (*n* tests)	*p*-value	q-value (BH)	Bonferroni-adjusted *p*-value
Correlation with VAS Neck (Table 4)				
SCL-27 Dysthymic Score	Psychological (13)	0.015	0.097	0.195
SCL-27 Vegetative Score	Psychological (13)	< 0.001	**0.006**	**0.007**
C1C2 lordosis (°)	Radiographic (7)	0.010	0.070	0.070
Pairwise comparison, VAS Neck ≤ 3 vs. ≥ 4 (Table 5)				
SCL-27 Dysthymic Score	Psychological (13)	0.006	**0.035**	0.078
SCL-27 Vegetative Score	Psychological (13)	< 0.001	**0.006**	**0.007**
SCL-27 Agoraphobic Score	Psychological (13)	0.029	0.094	0.377
SCL-27 Sociophobic Score	Psychological (13)	0.043	0.112	0.559
SCL-27 GSI	Psychological (13)	0.008	**0.035**	0.104
C1C2 lordosis (°)	Radiographic (7)	0.022	0.154	0.154
Pairwise comparison, VAS Neck ≤ 6 vs. ≥ 7 (Table 5)				
C1C2 lordosis (°)	Radiographic (7)	0.024	0.168	0.168
Univariate regression, VAS Neck as dependent variable (Table 6)				
SCL-27 Dysthymic Score	Psychological (13)	0.016	0.104	0.208
SCL-27 Vegetative Score	Psychological (13)	< 0.001	**0.006**	**0.007**
SCL-27 GSI	Psychological (13)	0.024	0.104	0.312

Correction was applied within two pre-specified hypothesis domains and separately for each analytical family (correlation analysis, each pairwise comparison, and univariate regression). The Benjamini-Hochberg procedure was used to control the false discovery rate at 0.05; Bonferroni-adjusted p-values are reported as a more conservative sensitivity check. Only variables reaching nominal significance (*p* < 0.05) are listed. *BH* Benjamini-hochberg, *GSI* Global severity index, *SCL-27* Symptom checklist-27, *I-TIPI* Ten-item personality inventory, *CD-RISC10* Connor-davidson resilience scale − 10 items, *VAS* Visual analogue scale. Bold font indicates statistical significance after Benjamini-Hochberg correction

In the multiple imputation sensitivity analysis, all estimates were consistent with the complete-case results, with a fraction of missing information not exceeding 0.12 for any variable; the associations of neck pain with the SCL-27 dysthymic and vegetative subscales were confirmed, and the association with the GSI reached significance in both the correlation and the regression analysis (Supplementary Table S1).

## Discussion

The pathophysiology of neck pain in cervical spondylosis has been investigated with a primary focus on mechanical factors secondary to age-related degenerative changes of structural elements. This predominantly mechanical perspective has led to controversy and inconsistent findings [2–4]. The present cohort study was based on the hypothesis that these inconsistencies arise from a non-comprehensive approach, and that psychological and adaptive factors mediating the perception of neck pain should also be considered. Previous studies addressing this issue have mainly focused on patients with a psychiatric history or have used non-specific tools that did not adequately capture the full range of psychological and adaptive strategies [9, 11]. In this study, we evaluated perceived neck pain in patients with cervical spondylosis without cervical deformity by considering both the mechanical factors previously associated with neck pain and a set of self-administered psychological questionnaires assessing personality, resilience and psychological symptoms. We found a significant correlation between perceived neck pain and SCL-27 dysthymic and vegetative subscales, as well as with C1–C2 lordosis. In contrast, no significant correlation was observed with personality and resilience test scores, nor with previously described sagittal alignment parameters, including T1S, cervical mismatch, and cSVA [2, 3]. After stratifying patients by neck pain severity, dysthymic, vegetative, agoraphobic, sociophobic, and GSI SCL-27 scores were significantly higher in the moderate-severe pain group than in the mild pain group. This pattern was not maintained from moderate to severe pain, where these scores were similar or even lower than in the moderate pain group. Conversely, C1–C2 lordosis showed a progressive and significant increase from the mild to the severe pain group. In univariate analysis, only the psychological symptom subscales (dysthymic, vegetative, and GSI) emerged as significant associations of perceived neck pain. Overall, the present cohort study is, to our knowledge, the first to highlight the relevance of dysthymic and vegetative psychological profiles in perceived neck pain among patients with non-deformative cervical spondylosis reporting moderate-severe neck pain, and to identify progressively increasing C1–C2 lordosis as a key factor in the transition from moderate to severe neck pain. These findings showed a remarkable robustness. After correction for multiple comparisons, the association between neck pain and SCL-27 vegetative symptoms was confirmed across all analyses, and the dysthymic and GSI scores retained significance in the comparison between mild and moderate-to-severe pain. Conversely, the association with C1–C2 lordosis did not survive correction in any analysis and should therefore be regarded as hypothesis-generating rather than established.

Psychological factors appear to contribute to the variability of neck pain perception [24–26]. Previous studies on cervical and lumbar degenerative spondylosis have consistently identified depression as a relevant negative determinant of clinical outcomes [9]. However, in the present study, depressive symptoms did not emerge as a significant factor in any of the performed analyses. Conversely, dysthymic symptoms showed a consistent association with pain-related outcomes. This finding may be explained by the different conceptual meaning of major depression as a categorical psychiatric diagnosis and dysthymia as a dimensional psychological trait. While major depression represents a clinically defined disorder, dysthymia reflects a persistent pattern of low mood, emotional fatigue, and negative affectivity that often remains below the diagnostic threshold of major depressive disorder [16, 27]. As such, dysthymia may represent a more sensitive marker of chronic psychological burden in patients with long-standing musculoskeletal disorders. In our cohort, a substantial proportion of patients exhibited dysthymic scores above normative values, despite the absence of a formal diagnosis of depression. This finding suggests that psychological vulnerability in patients with cervical spondylosis may be more accurately captured by subthreshold affective traits rather than by psychiatric disorders diagnosed according to clinical criteria. This may also explain why previous studies, relying primarily on documented depression, have reported heterogeneous results regarding the psychological determinants of neck pain. It should also be noted that the SCL-27 assesses the presence and severity of specific psychological symptoms. The depressive subscale includes items reflecting severe depressive content, such as thoughts of death or dying and suicidal ideation. The lack of significant findings on this dimension in our cohort therefore suggests that patients did not present with overt depressive symptomatology. This further supports the interpretation of a psychological profile characterized by subclinical affective symptoms rather than by major depressive features.

Vegetative symptoms also emerged as a significant correlate of perceived pain in our analysis. These symptoms, which include fatigue, sleep disturbance, and somatic discomfort, are commonly associated with chronic pain conditions and may contribute to pain amplification through sustained physiological and autonomic activation [28, 29]. In patients with degenerative cervical disease, such symptoms may interact with biomechanical stressors, contributing to increased pain perception independently of radiographic severity.

In contrast, neither resilience nor personality traits showed a significant association with neck pain in the present cohort. This result may be explained by the relatively homogeneous distribution of these variables, with most patients presenting values within normative ranges. Consequently, personality structure and global resilience may have limited discriminative power in a population without extreme psychological profiles. These findings suggest that, in patients with non-deformative cervical spondylosis, pain perception is more closely related to subtle affective symptoms than to stable personality traits or resilience levels.

Upper cervical lordosis (UCL) functions as a compensatory mechanism for variations in lower cervical lordosis, which in turn counteracts changes in thoracic alignment associated with age-related degeneration or degenerative deformities; overall, the system aims to maintain a horizontal gaze [5, 7, 30–32]. The biomechanical studies by Lee et al. and Le Huec et al. in two cohorts of asymptomatic volunteers reported the chain of correlations between the C0C2 angle and CL, and CL and T1S, whereas no correlation was found between C0C2 and T1S [5, 31]. After 50 years of age, C7S and T1S tend to increase in parallel with the progression of thoracic kyphosis. Consequently, the lordosis of the lower subaxial arc and UCL tends to increase in a compensatory manner. Again, no correlation was observed between CL and UCL [33]. In patients with spinal deformity, however, this pattern appears to change, underscoring the critical role of UCL in preserving horizontal gaze as a rescue strategy. Both Staub et al. and Protopsaltis et al. identified a strong positive correlation between UCL angles and cervical mismatch, indicating that UCL contributes to maintaining horizontal gaze when CL is insufficient to compensate for T1S, thereby further supporting the compensatory role of UCL in the setting of reduced CL [7, 32]. The results of our study should be interpreted within this mechanical framework, as we specifically included a population with cervical spondylosis without deformity. Our cohort showed only a modest increase in cervical mismatch with increasing perceived neck pain severity, characterized by a non-significant reduction in CL that exceeded that of T1S, together with a compensatory increase in upper CL. Previous studies on the mechanical implications of neck pain and related disability have mainly focused on populations with cervical deformity. Iver et al. reported a strong correlation between CL and T1S–CL only in the cervical deformity subgroup, whereas this association was not confirmed in the non-deformity subgroup, which was limited by a small sample size (*N* = 21) [2]. Similarly, Li et al. investigated a cohort with cervical kyphosis and identified cSVA and T1S as independent predictors of VAS neck ≥ 3, but did not consider cervical mismatch or UCL, thereby limiting the generalizability of their findings [3]. In this context, our results provide additional insight into neck pain in patients without cervical deformity, supported by a larger sample size (*N* = 130). Specifically, we found that modest reductions in CL and increases in cervical mismatch were not associated with perceived neck pain, whereas a compensatory increase in C1–C2 lordosis was related to higher reported neck pain. This association appeared particularly relevant in the group with the highest neck pain severity, in which no other investigated factors showed statistically significant association with neck pain.

### Limitations and perspectives

The generalizability of the present study is constrained by several relevant factors. First, there is a geographical limitation, as the study was conducted on an Italian sample, which may restrict the applicability of the results to other regions. The participants also displayed specific psychological characteristics, including low-to-moderate levels of psychological symptoms and comparatively high levels of agreeableness and conscientiousness relative to normative data, thereby limiting our conclusions to individuals with a relatively low psychological burden. Moreover, the focus on a degenerative, non-deformity population further reduces the generalizability of the findings. Future research is needed to address these limitations by including more psychologically distressed patients, recruiting samples from different geographical areas, and incorporating cervical spine deformity cases. Nonetheless, this study offers important future directions. It supports the need to investigate the impact of preoperative psychological rehabilitation on perceived neck pain. Second, it highlights the importance of examining how baseline psychological profiles influence postoperative outcomes in subsequent studies.

A further limitation concerns the completeness of the psychological assessment. Questionnaires were self-administered and were returned incomplete by a range from 10.0% to 15.0% of patients, and incompleteness increased monotonically with pain severity, so that patients with the greatest symptom burden are the least represented in the psychological analyses. The multiple imputation sensitivity analysis marginally strengthened the association with the GSI. The direction of this bias is therefore towards underestimation rather than inflation of the reported associations. This observation also carries a methodological implication for future work in this field, since compliance with psychological questionnaires appears to deteriorate precisely in the patients in whom psychological assessment is most informative.

Detailed clinical characterisation of the cohort was not available. The distribution of presenting symptoms (axial neck pain, radiculopathy, myelopathy), the duration of neck pain, the number of affected levels, the use of analgesic or psychotropic medication and the sex distribution could not be retrieved from the registry dataset. This is a substantial limitation and the associations we describe cannot be adjusted for them. The findings presented here should accordingly be regarded as unadjusted associations, and their confirmation requires further studies in which clinical phenotype, symptom chronicity and pharmacological treatment are prospectively recorded.

## Conclusion

The current study underscores the importance of psychological factors in shaping the perception of neck pain among individuals with non-deformative cervical spondylosis. Psychological symptoms measured with the SCL-27, particularly the vegetative and dysthymic subscales, were consistently associated with pain intensity and retained significance after correction for multiple comparisons, whereas no sagittal alignment parameter showed a robust association. Preoperative screening with the SCL-27 may help identify patients with a greater psychological symptom burden and may support individualised counselling. Whether such screening modifies clinical outcomes, and whether preoperative psychological support alters perceived neck pain, remain questions for prospective longitudinal investigation.

Included patients: 130.

## Supplementary information

Below is the link to the electronic supplementary material.

Supplementary Material 1

## Data Availability

No datasets were generated or analysed during the current study.

## Abbreviations

C2S: C2 slope
C7S: C7 slope
CD-RISC10: Connor-davidson resilience scale − 10 items
CL: C2C7 Cervical lordosis
cSVA: Cervical Sagittal vertical axis
I-TIPI: Ten-item personality inventory
MGS: McGregor slope
PANDA: Progressive advancement in neurosurgery: data and analysis
SCL-27: Symptom checklist-27
T1S: T1 slope
UCL: C1C2 cervical lordosis
VAS: Visual analogue scale

## Declarations

### Progressive advancement in neurosurgery: data and analysis (PANDA)

Registry: number NCT06835634, date of registration 15/01/2025.
