# Supplementary material for: A comprehensive analysis of the association of psychological profiling and sagittal balance parameters with neck pain in a non-deformity cervical spondylosis population: A prospective two-centers cross-sectional study
Source: Neurosurg Rev. 2026 Oct 19;49(1):617. doi: 10.1007/s10143-026-04527-z (PMC13677632; doi:10.1007/s10143-026-04527-z)
Supplement: Supplementary file 1 [file 10143_2026_4527_MOESM1_ESM.docx]

Supplementary Table S1. Sensitivity analysis: complete-case analysis versus multiple imputation of missing psychological data (m = 20).

| **Variable** | **Complete-case estimate** | **p-value** | **Pooled estimate after MI** | **p-value** | **FMI** |
| --- | --- | --- | --- | --- | --- |
| **Spearman correlation with VAS Neck (rho)** | | | | | |
| SCL-27 Depressive Score | 0.054 | 0.588 | 0.060 | 0.549 | 0.07 |
| **SCL-27 Dysthymic Score** | **0.239** | **0.015** | **0.208** | **0.037** | **0.09** |
| **SCL-27 Vegetative Score** | **0.344** | **<0.001** | **0.334** | **0.001** | **0.07** |
| SCL-27 Agoraphobic Score | 0.155 | 0.120 | 0.140 | 0.159 | 0.08 |
| SCL-27 Sociophobic Score | 0.079 | 0.437 | 0.091 | 0.363 | 0.08 |
| SCL-27 Mistrust Score | -0.056 | 0.578 | -0.030 | 0.764 | 0.06 |
| **SCL-27 GSI** | **0.198** | **0.053** | **0.213** | **0.030** | **0.06** |
| I-TIPI Extraversion | 0.014 | 0.889 | 0.009 | 0.925 | 0.04 |
| I-TIPI Agreeableness | -0.120 | 0.223 | -0.107 | 0.284 | 0.08 |
| I-TIPI Conscientiousness | -0.017 | 0.865 | -0.016 | 0.867 | 0.06 |
| I-TIPI Openness to Experience | 0.048 | 0.632 | 0.046 | 0.652 | 0.11 |
| I-TIPI Emotional Stability | -0.017 | 0.867 | -0.011 | 0.907 | 0.05 |
| CD-RISC-10 | -0.076 | 0.452 | -0.067 | 0.498 | 0.07 |
| **C1C2 lordosis (°)** | **-0.244** | **0.010** | **-0.254** | **0.008** | **0.01** |
| C2C7 lordosis (°) | 0.055 | 0.563 | 0.055 | 0.565 | 0.00 |
| T1S (°) | 0.006 | 0.948 | 0.006 | 0.948 | 0.00 |
| T1-CL (°) | 0.151 | 0.109 | 0.151 | 0.112 | 0.00 |
| cSVA (mm) | 0.076 | 0.424 | 0.076 | 0.427 | 0.00 |
| MGS (°) | -0.114 | 0.236 | -0.102 | 0.303 | 0.07 |
| C2S (°) | 0.136 | 0.151 | 0.136 | 0.154 | 0.00 |
| **Univariate linear regression, VAS Neck as dependent variable (ß-coefficient)** | | | | | |
| SCL-27 Depressive Score | 0.25 | 0.729 | 0.31 | 0.663 | 0.09 |
| **SCL-27 Dysthymic Score** | **0.99** | **0.016** | **0.88** | **0.026** | **0.07** |
| **SCL-27 Vegetative Score** | **1.81** | **<0.001** | **1.71** | **0.001** | **0.09** |
| SCL-27 Agoraphobic Score | 1.22 | 0.117 | 1.11 | 0.143 | 0.04 |
| SCL-27 Sociophobic Score | 0.52 | 0.402 | 0.58 | 0.331 | 0.06 |
| SCL-27 Mistrust Score | -0.03 | 0.954 | 0.10 | 0.847 | 0.06 |
| **SCL-27 GSI** | **1.81** | **0.024** | **1.86** | **0.015** | **0.04** |
| I-TIPI Extraversion | 0.03 | 0.895 | 0.02 | 0.935 | 0.03 |
| I-TIPI Agreeableness | -0.31 | 0.269 | -0.27 | 0.324 | 0.08 |
| I-TIPI Conscientiousness | -0.05 | 0.858 | -0.04 | 0.860 | 0.06 |
| I-TIPI Openness to Experience | 0.05 | 0.859 | 0.04 | 0.882 | 0.10 |
| I-TIPI Emotional Stability | -0.12 | 0.608 | -0.10 | 0.665 | 0.03 |
| CD-RISC-10 | -0.04 | 0.393 | -0.04 | 0.421 | 0.09 |
| C1C2 lordosis (°) | -0.06 | 0.094 | -0.06 | 0.072 | 0.01 |
| C2C7 lordosis (°) | 0.00 | 0.918 | 0.00 | 0.918 | 0.00 |
| T1S (°) | 0.01 | 0.677 | 0.01 | 0.677 | 0.00 |
| T1-CL (°) | 0.02 | 0.575 | 0.02 | 0.575 | 0.00 |
| cSVA (mm) | 0.01 | 0.530 | 0.01 | 0.530 | 0.00 |
| MGS (°) | -0.03 | 0.459 | -0.03 | 0.538 | 0.12 |
| C2S (°) | 0.04 | 0.254 | 0.04 | 0.254 | 0.00 |

Missing values in the psychological instruments and in the C1C2 lordosis and McGregor slope measurements were imputed by multivariate imputation by chained equations (m = 20), using all psychological, radiographic and demographic variables and the outcome (VAS Neck) as predictors; imputed values were constrained within the admissible range of each instrument. The Global Severity Index was derived passively from the imputed subscale scores rather than imputed directly. Estimates were pooled according to Rubin's rules; correlation coefficients were pooled on the Fisher z scale. Bold font indicates variables reaching statistical significance in either analysis. FMI: fraction of missing information; GSI: Global Severity Index; MI: multiple imputation; SCL-27: Symptom Checklist-27; I-TIPI: Ten-Item Personality Inventory; CD-RISC10: Connor-Davidson Resilience Scale - 10 items; T1S: T1 slope; cSVA: cervical sagittal vertical axis; C2S: C2 slope; MGS: McGregor slope; VAS: Visual Analogue Scale.
